# Personal experience: Diagnosis and dilemmas - what happens when we diagnose patients with the label ‘schizophrenia’

**DOI:** 10.1192/pb.bp.113.046631

**Published:** 2014-08

**Authors:** Mark Ellerby

At some point during the first onset of schizophrenia, family members are going to ask the question ‘What is wrong with him (or her)?’ The result is going to be a diagnosis or medical explanation in some form. Even today, with years of first-hand experience of schizophrenia, I am still unsure about how to best approach this thorny issue. The problem is that it is easy to get the wrong first impression from all the terms and labels that surround a mental illness. What follows here is an account of the mistaken initial impressions I formed once I was diagnosed and a hope that others who read this will not form the same misperceptions as I had.

The diagnosis can be bewildering for all concerned. My family was equally frightened and worried. As there had been some history of mental illness in the family, they knew that seeing a doctor was the right thing to do. They also had some notion of what a mental illness is. I was much less well informed. Many things about my first diagnosis confused me and mystified the whole subject of being ‘ill’. I had no grasp of the distinction between mental illness and physical illness or that I had a mental medical condition. When I was told it might be schizophrenia, the word to me as a layman was about as clear as mud. I was at a total loss as to what this could be. It never occurred to me that it was a form of madness, in fact, it initially rang no bells with me at all. But since a doctor had suggested this, it began to prey on my mind. I looked it up and found that there were four different types of schizophrenia. It did occur to me that in my case it was ‘paranoid schizophrenia’, but what ‘hebrephrenic’ and ‘simple’ meant I had no idea, and unfortunately the book did not elaborate. Again, in contrast to these latter types, the idea of being catatonic was readily intelligible. All the same, I asked the doctor about the mysterious classification just to make sure that, in my ignorance, I was putting myself in the right category, to make sure I fully understood what I was being labelled with and whether there were any aspects of the other types of schizophrenia I was experiencing that might have been relevant.

Much later, a community psychiatric nurse said that the fourfold typology is hardly used. In fact, she could hardly believe that I had heard of it at all and regarded it as antiquated. She emphasised that a little knowledge is a dangerous thing, a thought that did not occur to me at the time. Admittedly, the book did make the point that it literally meant ‘split mind’, which I took to mean a split personality. But in contrast to what it also said about being paranoid, this fact just confused even more. Did I have one part of my personality that was paranoid and the other normal as the delusions came and went, I thought? On the other hand, the word ‘psychotic’ turned out to be a very common medical expression. My initial thought here was ‘Is there a distinction between psychoses and neuroses?’, but once again my psychiatrist told me ‘I do not believe there is any such thing as a neurosis.’ After that I did not dare ask what psychotic was for fear of looking stupid.

So again, at the time I was mystified by the distinction, especially as it appeared in the same book on psychology I had looked schizophrenia up in. I assumed it was because it was a psychology textbook, not a psychiatric one, and that my psychiatrist’s response meant it was a technical dispute between the two professions.

The main impression I formed about all of this - when I got to thinking about it more - and that all these terms created for me was that there might be something dangerous about having schizophrenia. Maybe this was a sign of madness after all? I began to get worried. I got the feeling in the back of my mind that maybe I really was a little mad underneath. However, at no point did any professional discuss what my diagnosis meant. It was stated as fact, with no room for dialogue or understanding. I was left on my own to find out more but my reading made me anxious and it was difficult to think through what it all meant for me. What actually prevented me from giving these descriptions a fuller consideration was that I was too caught up with the delusions and voices I had begun hearing. I never got any rest from them and they were constantly on my mind. Hours seemed to fly by like minutes and in the end I lost all sense of the passage of time. Even when my mind had some opportunity to work on these concepts to do with mental health, and the other psychological terms, I still replied to the psychiatrist who came to visit me at home that I was not ill. By then I had absorbed the idea of having a mental illness, but that did not help my understanding of my situation, as I thought what I was thinking was real. I picked up the notion of illness from looking up medications in a library book and finding antipsychotics in it. Unfortunately, this connection made me even more suspicious of the term psychosis. Was the delusional part of myself seen as a danger by everyone else that needed to be ‘got rid of’? This thought caused more anxiety.

In sum, I think an effective explanation to me and my family would have made me more cooperative and could have been instrumental in getting me help sooner. The reason for this is that it is possible to form all kinds of misconceptions about schizophrenia and psychiatry that, if dispelled, could make a lot of difference.

Only in recent years have I been helped to find a personal formulation of my difficulties. This is a framework for understanding the personal triggering and maintaining factors in each person’s mental health issues. I have learnt that not everyone with my diagnosis is the same and that my history and personal experiences may have shaped the development of my problems.

## First contacts with a psychiatrist

There may be some resistance on the part of the patient to being interviewed. Why should I tell you about my problems? It is necessary to somehow get across the concept of being mentally ill and that people want to help you with it, not to lock you up.

Hospital design is part of the process. In the one I attend, there is coffee available, music playing and art on the walls. The informality of the staff is also important. Plus, pay attention to how psychiatrists and nurses dress - casually? First impressions, or are we too ill to notice? What more can be done to get the message across? Is it all in vain?

The answer here is no. I think people who constantly care for emotionally distressed people - nurses and doctors - demonstrate an ethos of care that manifests itself in their being and behaviour. This, in my hospital, is so visible and you can see the concern for patients’ well-being in eyes of the nurses; the above considerations seem to fade into the background. If the patient closes themselves off to the staff, a relationship of trust and confidence will be harder to establish. You might, if you are not aware of these influences (e.g. because you are so caught up by the illness), feel like you are just a part of the system rather than an individual in the eyes of the staff. This is not helpful.

If I were more aware of the buildings, I would have wondered a little about the notorious carceral history of psychiatry. In particular, the example of Bedlam: the magnificent buildings, the awful stories. Although I am doubtful newer buildings would help in this respect (they would just make me think of American sanatoriums).

One important point is that doctors and psychiatrists should know what they are doing when they label someone with ‘schizophrenia’. Having a social worker on hand to explain about the stigma in the media and the connotations the word has, the negative language involved and how it is all just ignorance in that respect would make a huge difference. This is better than just saying it is a chemical imbalance, which may be enlightening only if we are properly educated in this respect and can appreciate that the problems we are experiencing are chemically caused, rather than something that is just happening to us, and that this chemistry really is an illness. My parents also felt there was a general lack of information about the subject. We educate teenagers about war in schools, so why not schizophrenia? It is just about as frightening. In the end, some kind of public programme is needed to underpin initial psychiatric contact. This happens in Norway, where huge public awareness events, like schizophrenia days in Stavanger, give school leavers information from primarily young people who had received help for mental health issues.

People who cope with particularly severe schizophrenia should be awarded the Victoria Cross. This is the best public defence against stigma. However, the illness destroys what could be a fertile mind, to the great loss of our society. There are examples of high achievers, such John Nash, played in the film *A Beautiful Mind* by Russell Crowe, who have contributed much to the society that excludes them, and it is popularly known that genius can be linked to madness. A more compassionate society could benefit from these contributions and function far better for the welfare for its members, many of whom are likely to have mental health problems at some point in their lives. The world would be a far happier and more creative place if it were more compassionate.

## Changing the labels?

As I have stated, I was so confused by all the terms that surround having a mental illness and the wrongful connotations some of them have, I really had no idea what was going on, even when the doctor diagnosed me. This, to me, raises the question of whether changing the labels would make the whole confusing problems of being schizophrenic any clearer from the outset? Ultimately, I do not think the word ‘schizophrenia’ should be used at all. It may be better to simply describe it as ‘paranoia’ and hearing voices as ‘hallucinating’. These terms are not ideal, but they are far more innocuous and understandable than ‘schizophrenia’ or ‘psychosis’. In the end, you cannot avoid the confusions of explaining the illness without being careful about exactly what you say, and what you might leave out. ‘Schizophrenia’ is a real umbrella term, covering a number of different symptoms and indeed types of illness. It may be better to divide the diagnosis up on this basis, though often the different symptoms are experienced by the same individual. It has also been shown by research that the biology involved is linked to previous stress and trauma for many. I have often thought that the best thing to say instead of schizophrenia is that ‘you are hallucinating’ or ‘you are having delusions’. Although both of these are far from perfect, they do not have the associations of the label schizophrenia, which a lot of people would immediately connect with a split personality and Dr Jekyll and Mr Hyde.

Hallucinations are often associated with phenomena such as delirium. This happens when someone has a fever, and I have seen this in a couple of movies too. Hearing voices is often triggered by a loss and is common even in people who are outside the mental health system. I think it is better to start with everyday explanations, not just medical ones.

So this might be a useful first explanatory link and might avoid the first associations with schizophrenia. This is already how I have seen doctors technically denote what lay-people call hearing voices, which they term ‘auditory hallucinations’. Hallucinating also sounds a lot better and much less dangerous to me than saying someone hears voices. I am aware that many patient groups would disagree with what I have said here because hearing voices is so common and uses everyday language. However, ‘hallucinations’ only suggests another more medical-sounding label, which still may not be readily understood, but carries less associations of stigma.

Being ‘delusional’ again sounds a lot better to me than having schizophrenia. Delusions are in popular knowledge associated with insanity, such as ‘delusions of grandeur’. The immediate reaction here might even be humorous, as such ideas seem comical and absurd to the layman. ‘Paranoid delusions’ is another close association with schizophrenia but in this respect it could be a very counterproductive label. Again, I think there is a suggestion of danger associated with someone who is paranoid about people persecuting them. It is hard to see a way past this problem by simply using new terminology. What label would you suggest for people who believe others are out to get them? It might be helpful to try to give an example here of famous people who have had the same problem. There are lots of popular films, such as *The Madness of King George* and *A Beautiful Mind*. This seems the best way to defuse the connotations which arise from an initial diagnosis.

In the end, the whole issue of how to explain it seems very difficult. Normalising some of the experiences is critical. Being open about discussing people’s misconceptions and fears would help prevent those fears escalating. Giving good information to the patient and the family about options for recovery is critical in inspiring hope. Understanding each patient’s personal journey into psychosis would be more meaningful than blanket labels. The media has an important role to play in perpetuating stereotypes, which increase fears of the diagnosis of schizophrenia. Finally, raising mental health awareness with young people in a non-shaming way would give them the information they need to recognise issues early on and the courage to ask for support.

